# (*E*)-2-(3-Cinnamoyl­thio­ureido)acetic acid dimethyl sulfoxide disolvate

**DOI:** 10.1107/S1600536811036750

**Published:** 2011-09-17

**Authors:** Ibrahim N. Hassan, Wan Ramli Wan Daud, Bohari M. Yamin, Mohammad B. Kassim

**Affiliations:** a Fuel Cell Institute, Universiti Kebangsaan Malaysia, UKM 43600 Bangi Selangor, Malaysia; b Department of Chemical and Process Engineering, Faculty of Engineering, Universiti Kebangsaan Malaysia, UKM 43600 Bangi Selangor, Malaysia; c School of Chemical Sciences and Food Technology, Faculty of Science and Technology, Universiti Kebangsaan Malaysia, UKM 43600 Bangi Selangor, Malaysia

## Abstract

In the title compound, C_12_H_12_N_2_O_3_S·2C_2_H_6_OS, the acetic acid and cinnamoyl groups adopt *Z* and *E* configurations, respectively, with respect to the thio group about the C—N bonds. The components of the asymmetric unit are connected by N—H⋯O and O—H⋯O hydrogen bonds and in the crystal weak inter­molecular C—H⋯O and C—H⋯S hydrogen bonds further connect the components into chains along the *b* axis. In the main mol­ecule, an intra­molecular N—H⋯O hydrogen bond is also present.

## Related literature

For related structures, see: Hassan *et al.* (2009 ▶, 2010*a* ▶,*b* ▶,*c* ▶, 2011 ▶); Nasir *et al.* (2011 ▶). For the synthesis, see: Hassan *et al.* (2008 ▶). For standard bond-length data, see: Allen *et al.* (1987 ▶).
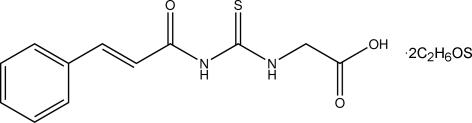


## Experimental

### 

#### Crystal data

C_12_H_12_N_2_O_3_S·2C_2_H_6_OS
*M* *_r_* = 420.55
Triclinic, 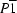

*a* = 7.327 (3) Å
*b* = 12.064 (5) Å
*c* = 13.691 (6) Å
α = 65.794 (8)°
β = 75.603 (9)°
γ = 85.484 (9)°
*V* = 1068.6 (8) Å^3^
*Z* = 2
Mo *K*α radiation
μ = 0.37 mm^−1^
*T* = 298 K
0.42 × 0.21 × 0.18 mm

#### Data collection

Bruker SMART APEX CCD diffractometer
Absorption correction: multi-scan (*SADABS*; Sheldrick, 1996 ▶) *T*_min_ = 0.859, *T*_max_ = 0.936
10964 measured reflections
3774 independent reflections
2650 reflections with *I* > 2σ(*I*)
*R*_int_ = 0.046

#### Refinement

*R*[*F* ^2^ > 2σ(*F* ^2^)] = 0.085
*wR*(*F* ^2^) = 0.251
*S* = 1.06
3774 reflections
240 parameters
H-atom parameters constrained
Δρ_max_ = 0.72 e Å^−3^
Δρ_min_ = −0.25 e Å^−3^

### 

Data collection: *SMART* (Bruker, 2000 ▶); cell refinement: *SAINT* (Bruker, 2000 ▶); data reduction: *SAINT*; program(s) used to solve structure: *SHELXS97* (Sheldrick, 2008 ▶); program(s) used to refine structure: *SHELXL97* (Sheldrick, 2008 ▶); molecular graphics: *ORTEPIII* (Burnett & Johnson, 1996 ▶) and *SHELXTL* (Sheldrick, 2008 ▶); software used to prepare material for publication: *SHELXTL* and *PLATON* (Spek, 2009 ▶).

## Supplementary Material

Crystal structure: contains datablock(s) global, I. DOI: 10.1107/S1600536811036750/lh5324sup1.cif

Structure factors: contains datablock(s) I. DOI: 10.1107/S1600536811036750/lh5324Isup2.hkl

Supplementary material file. DOI: 10.1107/S1600536811036750/lh5324Isup3.cml

Additional supplementary materials:  crystallographic information; 3D view; checkCIF report

## Figures and Tables

**Table 1:** Hydrogen-bond geometry (Å, °)

*D*—H⋯*A*	*D*—H	H⋯*A*	*D*⋯*A*	*D*—H⋯*A*
N1—H1*A*⋯O5	0.86	2.02	2.867 (5)	167
N2—H2*A*⋯O1	0.86	1.94	2.625 (4)	136
O3—H3*A*⋯O4	0.82	1.76	2.562 (5)	166
C14—H14*C*⋯O2	0.96	2.54	3.423 (8)	153
C14—H14*A*⋯O5^i^	0.96	2.43	3.319 (7)	154
C15—H15*B*⋯O4^ii^	0.96	2.58	3.468 (7)	153
C16—H16*B*⋯S1^iii^	0.96	2.85	3.702 (7)	148

Symmetry codes: (i) 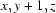; (ii) 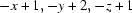; (iii) 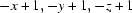.

**Fig. 1.**
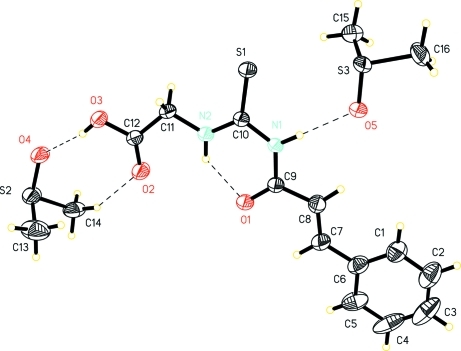
The molecular structure of (I), with displacement ellipsoids are drawn at the 50% probability level. Hydrogen bonds are shown as dashed lines.

**Fig. 2.**
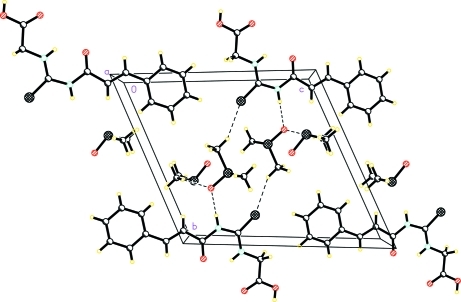
A packing diagram of (I) viewed along the c axis. Hydrogen bonds are shown by dashed lines.

## supplementary crystallographic information

### Comment

The title compound (I) is the thiourea carboxylic acid version of our previously reported ester molecules *(E)*-methyl-2-(3-cinnamoylthioureido)acetate (II) (Hassan *et al.*, 2010*a*)and *(E)*-ethyl-2-(3- cinnmoylthioureido)acetate (III) (Hassan *et al.*, 2010*b*) and analogous to methyl-2-(3-benzoylthioureido)acetate (IV) (Hassan *et al.*, 2009). The molecule maintains the same *E*-*Z* configuration with respect to the positions of the acetic acid and cinnamoyl groups, relative to the S atom across the C—N bonds, respectively (Fig. 1). In general, the bond lengths (Allen *et al.*, 1987) and angles in (I) are in normal ranges and comparable to those in (II), (III) and (IV). The the C═S bond length [1.665 (4) Å] is the same within experimental error to that in (II) [1.666 (3) Å] and that of (III) [1.656 (5) Å]. The C7═C8 bond length [1.331 (6) Å] is slightly longer than that reported by Hassan *et al.* (2010*c*) [1.320 (3) Å]. The carbonyl C═O bond length [1.215 (5) Å] is the same within experimental error to that reported by Nasir *et al.* (2011) [1.213 (3) Å]. The S1/O1/O2/N1/N2/C1—C12 fragment is essentially planar with a maximum deviation of 0.043 (5) Å, for atom C7. In the crystal, the components of the asymmtric unit are connected by N—H···O and O—H···O hydrogen bonds and weak intermolecular C—H···O and C—H···S hydrogen bonds connect the components into one-dimensional chains along the *b* axis (Fig. 2). In the main molecule an intramolecular N—H···O hydrogen bond is also present.

### Experimental

The title compound was synthesized according to a previously reported method (Hassan *et al.*, 2008). A yellowish crystal, suitable for X-ray crystallography, was obtained by a slow evaporation from CH_2_Cl_2_ solution at room temperature (yield 79%).

### Refinement

All H atoms were positioned geometrically and allowed to ride on their parent atoms with C—H = 0.93-0.97Å, N—H = 0.86Å and O—H = 0.82Å and with U_iso_(H) = 1.2U_eq_(C,N) or 1.5U_eq_(C_methyl_,O).

### Crystal data

**Table:**

C_12_H_12_N_2_O_3_S·2C_2_H_6_OS	*Z* = 2
*M**_r_* = 420.55	*F*(000) = 444
Triclinic, *P*1	*D*_x_ = 1.307 Mg m^−^^3^
Hall symbol: -P 1	Melting point: 407 K
*a* = 7.327 (3) Å	Mo *K*α radiation, λ = 0.71073 Å
*b* = 12.064 (5) Å	θ = 1.7–25.0°
*c* = 13.691 (6) Å	µ = 0.37 mm^−^^1^
α = 65.794 (8)°	*T* = 298 K
β = 75.603 (9)°	Block, colourless
γ = 85.484 (9)°	0.42 × 0.21 × 0.18 mm
*V* = 1068.6 (8) Å^3^	

### Data collection

**Table:**

Bruker SMART APEX CCD diffractometer	3774 independent reflections
Radiation source: fine-focus sealed tube	2650 reflections with *I* > 2σ(*I*)
graphite	*R*_int_ = 0.046
ω scans	θ_max_ = 25.0°, θ_min_ = 1.7°
Absorption correction: multi-scan (*SADABS*; Sheldrick, 1996)	*h* = −8→8
*T*_min_ = 0.859, *T*_max_ = 0.936	*k* = −14→14
10964 measured reflections	*l* = −16→16

### Refinement

**Table:**

Refinement on *F*^2^	Primary atom site location: structure-invariant direct methods
Least-squares matrix: full	Secondary atom site location: difference Fourier map
*R*[*F*^2^ > 2σ(*F*^2^)] = 0.085	Hydrogen site location: inferred from neighbouring sites
*wR*(*F*^2^) = 0.251	H-atom parameters constrained
*S* = 1.06	*w* = 1/[σ^2^(*F*_o_^2^) + (0.1544*P*)^2^ + 0.4047*P*] where *P* = (*F*_o_^2^ + 2*F*_c_^2^)/3
3774 reflections	(Δ/σ)_max_ < 0.001
240 parameters	Δρ_max_ = 0.72 e Å^−^^3^
0 restraints	Δρ_min_ = −0.25 e Å^−^^3^

### Special details

**Table:**

Geometry. All e.s.d.'s (except the e.s.d. in the dihedral angle between two l.s. planes) are estimated using the full covariance matrix. The cell e.s.d.'s are taken into account individually in the estimation of e.s.d.'s in distances, angles and torsion angles; correlations between e.s.d.'s in cell parameters are only used when they are defined by crystal symmetry. An approximate (isotropic) treatment of cell e.s.d.'s is used for estimating e.s.d.'s involving l.s. planes.
Refinement. Refinement of *F*^2^ against ALL reflections. The weighted *R*-factor *wR* and goodness of fit *S* are based on *F*^2^, conventional *R*-factors *R* are based on *F*, with *F* set to zero for negative *F*^2^. The threshold expression of *F*^2^ > σ(*F*^2^) is used only for calculating *R*-factors(gt) *etc*. and is not relevant to the choice of reflections for refinement. *R*-factors based on *F*^2^ are statistically about twice as large as those based on *F*, and *R*- factors based on ALL data will be even larger.

### Fractional atomic coordinates and isotropic or equivalent isotropic displacement parameters (Å^2^)

**Table:**

	*x*	*y*	*z*	*U*_iso_*/*U*_eq_	
S1	0.4518 (2)	0.84669 (10)	0.40846 (9)	0.0689 (5)	
S2	0.3003 (3)	1.64039 (11)	0.16460 (11)	0.0800 (6)	
S3	0.66629 (18)	0.58770 (10)	0.36710 (9)	0.0580 (4)	
O1	0.6554 (5)	1.1084 (3)	0.0515 (2)	0.0642 (10)	
O2	0.4605 (6)	1.3125 (3)	0.1829 (3)	0.0754 (11)	
O3	0.2971 (6)	1.3022 (3)	0.3467 (3)	0.0697 (11)	
H3A	0.3008	1.3765	0.3147	0.105*	
O4	0.2660 (7)	1.5335 (3)	0.2747 (3)	0.0836 (13)	
O5	0.7343 (5)	0.6780 (3)	0.2502 (2)	0.0626 (9)	
N1	0.6120 (5)	0.9232 (3)	0.1959 (3)	0.0457 (9)	
H1A	0.6309	0.8470	0.2128	0.055*	
N2	0.4931 (5)	1.0710 (3)	0.2566 (3)	0.0495 (9)	
H2A	0.5327	1.1210	0.1891	0.059*	
C1	0.9539 (8)	0.8304 (6)	−0.1493 (5)	0.0762 (16)	
H1	0.9122	0.7733	−0.0774	0.091*	
C2	1.0432 (9)	0.7939 (8)	−0.2298 (6)	0.097 (2)	
H2	1.0635	0.7115	−0.2118	0.116*	
C3	1.1037 (10)	0.8736 (11)	−0.3359 (8)	0.116 (3)	
H3	1.1623	0.8466	−0.3903	0.139*	
C4	1.0773 (10)	0.9927 (10)	−0.3607 (5)	0.105 (3)	
H4	1.1197	1.0486	−0.4331	0.126*	
C5	0.9890 (8)	1.0335 (7)	−0.2812 (4)	0.0843 (18)	
H5	0.9724	1.1163	−0.2998	0.101*	
C6	0.9249 (7)	0.9513 (5)	−0.1739 (4)	0.0648 (13)	
C7	0.8281 (7)	0.9974 (5)	−0.0920 (4)	0.0618 (13)	
H7A	0.8061	1.0803	−0.1186	0.074*	
C8	0.7671 (7)	0.9352 (4)	0.0163 (4)	0.0608 (13)	
H8A	0.7811	0.8514	0.0475	0.073*	
C9	0.6774 (6)	0.9993 (4)	0.0869 (3)	0.0492 (11)	
C10	0.5195 (6)	0.9548 (4)	0.2819 (3)	0.0444 (10)	
C11	0.4015 (7)	1.1193 (4)	0.3357 (3)	0.0491 (11)	
H11A	0.4714	1.0991	0.3921	0.059*	
H11B	0.2754	1.0838	0.3711	0.059*	
C12	0.3910 (6)	1.2548 (4)	0.2786 (3)	0.0492 (11)	
C13	0.1632 (10)	1.6099 (7)	0.0894 (5)	0.106 (2)	
H13A	0.1832	1.5281	0.0948	0.159*	
H13B	0.1992	1.6657	0.0134	0.159*	
H13C	0.0324	1.6191	0.1188	0.159*	
C14	0.5210 (8)	1.6206 (5)	0.0879 (4)	0.0731 (15)	
H14A	0.6180	1.6301	0.1201	0.110*	
H14B	0.5404	1.6803	0.0134	0.110*	
H14C	0.5254	1.5407	0.0884	0.110*	
C15	0.8192 (9)	0.6064 (6)	0.4402 (4)	0.0790 (16)	
H15A	0.9473	0.6011	0.4033	0.118*	
H15B	0.7928	0.5438	0.5136	0.118*	
H15C	0.8011	0.6846	0.4436	0.118*	
C16	0.7496 (11)	0.4446 (5)	0.3663 (5)	0.093 (2)	
H16A	0.6882	0.4229	0.3220	0.139*	
H16B	0.7219	0.3834	0.4403	0.139*	
H16C	0.8833	0.4507	0.3360	0.139*	

### Atomic displacement parameters (Å^2^)

**Table:**

	*U*^11^	*U*^22^	*U*^33^	*U*^12^	*U*^13^	*U*^23^
S1	0.1061 (11)	0.0398 (7)	0.0338 (6)	0.0062 (6)	0.0081 (6)	−0.0032 (5)
S2	0.1332 (14)	0.0414 (7)	0.0428 (7)	0.0122 (7)	−0.0026 (7)	−0.0070 (6)
S3	0.0743 (8)	0.0416 (6)	0.0401 (6)	0.0072 (5)	−0.0023 (5)	−0.0063 (5)
O1	0.101 (3)	0.0401 (17)	0.0329 (16)	0.0086 (16)	−0.0024 (16)	−0.0054 (13)
O2	0.125 (3)	0.0399 (17)	0.0402 (19)	0.0064 (18)	0.0049 (19)	−0.0102 (15)
O3	0.115 (3)	0.0444 (18)	0.0399 (17)	0.0144 (19)	−0.0037 (18)	−0.0176 (15)
O4	0.148 (4)	0.0432 (19)	0.0376 (18)	0.017 (2)	0.000 (2)	−0.0100 (15)
O5	0.100 (3)	0.0400 (16)	0.0360 (17)	0.0079 (16)	−0.0103 (16)	−0.0081 (14)
N1	0.069 (2)	0.0281 (16)	0.0323 (17)	0.0028 (15)	−0.0075 (16)	−0.0075 (14)
N2	0.071 (2)	0.0331 (18)	0.0317 (18)	0.0024 (16)	−0.0019 (16)	−0.0070 (14)
C1	0.076 (4)	0.087 (4)	0.063 (3)	0.011 (3)	−0.005 (3)	−0.036 (3)
C2	0.079 (4)	0.133 (6)	0.102 (6)	0.017 (4)	−0.012 (4)	−0.079 (5)
C3	0.078 (5)	0.205 (10)	0.105 (6)	−0.006 (6)	0.001 (4)	−0.114 (7)
C4	0.087 (5)	0.180 (9)	0.043 (3)	−0.043 (5)	0.013 (3)	−0.049 (5)
C5	0.083 (4)	0.105 (5)	0.041 (3)	−0.022 (3)	0.010 (3)	−0.016 (3)
C6	0.061 (3)	0.083 (4)	0.048 (3)	0.002 (3)	−0.005 (2)	−0.028 (3)
C7	0.080 (3)	0.057 (3)	0.040 (2)	0.002 (2)	−0.005 (2)	−0.016 (2)
C8	0.080 (3)	0.044 (3)	0.046 (3)	0.002 (2)	−0.002 (2)	−0.014 (2)
C9	0.066 (3)	0.038 (2)	0.034 (2)	0.0030 (19)	−0.004 (2)	−0.0099 (18)
C10	0.058 (3)	0.036 (2)	0.035 (2)	0.0030 (18)	−0.0094 (19)	−0.0114 (17)
C11	0.065 (3)	0.044 (2)	0.030 (2)	0.004 (2)	−0.0035 (19)	−0.0121 (18)
C12	0.068 (3)	0.044 (2)	0.035 (2)	0.006 (2)	−0.011 (2)	−0.0163 (19)
C13	0.091 (5)	0.121 (6)	0.063 (4)	−0.011 (4)	−0.010 (3)	0.004 (4)
C14	0.095 (4)	0.069 (3)	0.042 (3)	−0.011 (3)	−0.011 (3)	−0.010 (2)
C15	0.097 (4)	0.079 (4)	0.049 (3)	0.020 (3)	−0.015 (3)	−0.019 (3)
C16	0.130 (5)	0.037 (3)	0.076 (4)	0.007 (3)	0.008 (4)	−0.007 (3)

### Geometric parameters (Å, °)

**Table:**

S1—C10	1.664 (4)	C4—C5	1.372 (9)
S2—O4	1.506 (3)	C4—H4	0.9300
S2—C14	1.754 (6)	C5—C6	1.378 (7)
S2—C13	1.757 (7)	C5—H5	0.9300
S3—O5	1.501 (3)	C6—C7	1.457 (7)
S3—C15	1.758 (6)	C7—C8	1.331 (6)
S3—C16	1.789 (6)	C7—H7A	0.9300
O1—C9	1.215 (5)	C8—C9	1.477 (6)
O2—C12	1.199 (5)	C8—H8A	0.9300
O3—C12	1.313 (5)	C11—C12	1.500 (6)
O3—H3A	0.8200	C11—H11A	0.9700
N1—C9	1.375 (5)	C11—H11B	0.9700
N1—C10	1.386 (5)	C13—H13A	0.9600
N1—H1A	0.8600	C13—H13B	0.9600
N2—C10	1.310 (5)	C13—H13C	0.9600
N2—C11	1.434 (5)	C14—H14A	0.9600
N2—H2A	0.8600	C14—H14B	0.9600
C1—C2	1.353 (8)	C14—H14C	0.9600
C1—C6	1.368 (8)	C15—H15A	0.9600
C1—H1	0.9300	C15—H15B	0.9600
C2—C3	1.353 (12)	C15—H15C	0.9600
C2—H2	0.9300	C16—H16A	0.9600
C3—C4	1.344 (13)	C16—H16B	0.9600
C3—H3	0.9300	C16—H16C	0.9600
			
O4—S2—C14	106.8 (3)	O1—C9—C8	123.3 (4)
O4—S2—C13	105.6 (3)	N1—C9—C8	113.7 (4)
C14—S2—C13	97.0 (3)	N2—C10—N1	116.2 (3)
O5—S3—C15	105.7 (3)	N2—C10—S1	124.2 (3)
O5—S3—C16	104.9 (2)	N1—C10—S1	119.6 (3)
C15—S3—C16	97.7 (3)	N2—C11—C12	109.5 (3)
C12—O3—H3A	109.5	N2—C11—H11A	109.8
C9—N1—C10	127.7 (3)	C12—C11—H11A	109.8
C9—N1—H1A	116.1	N2—C11—H11B	109.8
C10—N1—H1A	116.1	C12—C11—H11B	109.8
C10—N2—C11	123.4 (3)	H11A—C11—H11B	108.2
C10—N2—H2A	118.3	O2—C12—O3	124.4 (4)
C11—N2—H2A	118.3	O2—C12—C11	124.1 (4)
C2—C1—C6	119.9 (6)	O3—C12—C11	111.5 (4)
C2—C1—H1	120.0	S2—C13—H13A	109.5
C6—C1—H1	120.0	S2—C13—H13B	109.5
C1—C2—C3	122.0 (8)	H13A—C13—H13B	109.5
C1—C2—H2	119.0	S2—C13—H13C	109.5
C3—C2—H2	119.0	H13A—C13—H13C	109.5
C4—C3—C2	118.5 (6)	H13B—C13—H13C	109.5
C4—C3—H3	120.8	S2—C14—H14A	109.5
C2—C3—H3	120.8	S2—C14—H14B	109.5
C3—C4—C5	121.3 (7)	H14A—C14—H14B	109.5
C3—C4—H4	119.4	S2—C14—H14C	109.5
C5—C4—H4	119.4	H14A—C14—H14C	109.5
C4—C5—C6	119.7 (7)	H14B—C14—H14C	109.5
C4—C5—H5	120.1	S3—C15—H15A	109.5
C6—C5—H5	120.1	S3—C15—H15B	109.5
C1—C6—C5	118.5 (5)	H15A—C15—H15B	109.5
C1—C6—C7	123.1 (5)	S3—C15—H15C	109.5
C5—C6—C7	118.4 (5)	H15A—C15—H15C	109.5
C8—C7—C6	127.9 (5)	H15B—C15—H15C	109.5
C8—C7—H7A	116.0	S3—C16—H16A	109.5
C6—C7—H7A	116.0	S3—C16—H16B	109.5
C7—C8—C9	119.9 (4)	H16A—C16—H16B	109.5
C7—C8—H8A	120.0	S3—C16—H16C	109.5
C9—C8—H8A	120.0	H16A—C16—H16C	109.5
O1—C9—N1	122.9 (4)	H16B—C16—H16C	109.5
			
C6—C1—C2—C3	−0.9 (10)	C10—N1—C9—O1	−2.1 (8)
C1—C2—C3—C4	1.5 (12)	C10—N1—C9—C8	−179.0 (4)
C2—C3—C4—C5	−0.8 (12)	C7—C8—C9—O1	−0.2 (8)
C3—C4—C5—C6	−0.3 (11)	C7—C8—C9—N1	176.6 (4)
C2—C1—C6—C5	−0.2 (9)	C11—N2—C10—N1	−179.4 (4)
C2—C1—C6—C7	179.3 (6)	C11—N2—C10—S1	−0.1 (7)
C4—C5—C6—C1	0.8 (9)	C9—N1—C10—N2	−0.9 (7)
C4—C5—C6—C7	−178.7 (6)	C9—N1—C10—S1	179.8 (4)
C1—C6—C7—C8	5.5 (9)	C10—N2—C11—C12	−178.7 (4)
C5—C6—C7—C8	−175.0 (6)	N2—C11—C12—O2	−4.4 (7)
C6—C7—C8—C9	177.8 (5)	N2—C11—C12—O3	175.8 (4)

### Hydrogen-bond geometry (Å, °)

**Table:**

*D*—H···*A*	*D*—H	H···*A*	*D*···*A*	*D*—H···*A*
N1—H1A···O5	0.86	2.02	2.867 (5)	167.
N2—H2A···O1	0.86	1.94	2.625 (4)	136.
O3—H3A···O4	0.82	1.76	2.562 (5)	166.
C14—H14C···O2	0.96	2.54	3.423 (8)	153
C14—H14A···O5^i^	0.96	2.43	3.319 (7)	154
C15—H15B···O4^ii^	0.96	2.58	3.468 (7)	153
C16—H16B···S1^iii^	0.96	2.85	3.702 (7)	148

Symmetry codes: (i) *x*, *y*+1, *z*; (ii) −*x*+1, −*y*+2, −*z*+1; (iii) −*x*+1, −*y*+1, −*z*+1.

## References

[bb1] Allen, F. H., Kennard, O., Watson, D. G., Brammer, L., Orpen, A. G. & Taylor, R. (1987). *J. Chem. Soc. Perkin Trans. 2*, pp. S1–19.

[bb2] Bruker (2000). *SMART* and *SAINT* Bruker AXS Inc., Madison, Wisconsin, USA.

[bb3] Burnett, M. N. & Johnson, C. K. (1996). *ORTEPIII* Report ORNL-6895. Oak Ridge National Laboratory, Tennessee, USA.

[bb4] Hassan, I. N., Yamin, B. M. & Kassim, M. B. (2008). *Acta Cryst.* E**64**, o1727. 10.1107/S1600536808024896 PMC2960614 21201710

[bb5] Hassan, I. N., Yamin, B. M. & Kassim, M. B. (2009). *Acta Cryst.* E**65**, o3078. 10.1107/S1600536809046169 PMC2972120 21578808

[bb6] Hassan, I. N., Yamin, B. M. & Kassim, M. B. (2010*a*). *Acta Cryst.* E**66**, o2242. 10.1107/S1600536810030084 PMC3007976 21588609

[bb7] Hassan, I. N., Yamin, B. M. & Kassim, M. B. (2010*b*). *Acta Cryst.* E**66**, o2784. 10.1107/S1600536810037918 PMC3009106 21588982

[bb8] Hassan, I. N., Yamin, B. M. & Kassim, M. B. (2010*c*). *Acta Cryst.* E**66**, o2796. 10.1107/S1600536810040018 PMC3009376 21588993

[bb9] Hassan, I. N., Yi, C. Y. & Kassim, M. B. (2011). *Acta Cryst.* E**67**, o780. 10.1107/S160053681100568X PMC3100062 21754071

[bb10] Nasir, M. F. M., Hassan, I. N., Wan Daud, W. R., Yamin, B. M. & Kassim, M. B. (2011). *Acta Cryst.* E**67**, o1987. 10.1107/S1600536811026687 PMC3213443 22091022

[bb11] Sheldrick, G. M. (1996). *SADABS* University of Göttingen, Germany.

[bb12] Sheldrick, G. M. (2008). *Acta Cryst.* A**64**, 112–122. 10.1107/S0108767307043930 18156677

[bb13] Spek, A. L. (2009). *Acta Cryst.* D**65**, 148–155. 10.1107/S090744490804362X PMC2631630 19171970

